# Microscopic Preparations

**Published:** 1853-09

**Authors:** 


### *Microscopic Preparations.*

To the Editor of the Boston Medical and Surgical Journal.

I send you a page or two relating to microscopic matters, which some of the students who read your Journal may like to see, if you can find room for so much in any of your coming numbers.

Yours very truly, O. W. Holmes.

Many of the readers of this Journal, and especially many of its younger readers, are interested in the microscope in its application to anatomy, physiology, and pathology. Most of the young physicians who complete their studies in Europe, bring home a "Nachet," or an "Oberhaeuser," and a certain amount of skill in handling it, which they find abundant leisure to improve in the early times of their practice. There are now many good instruments among us in the hands of those who know how to use them, and several of the highest excellence. Our microscopists are beginning to be known somewhat beyond their own immediate circle. Dr. Dalton and Dr. Burnett have been honored by two of the four prizes conferred by the American Medical Association, for essays based in great part or wholly on microscopic investigations. Other observers are at work, who will be heard from in due season.

In the mean time attention has been drawn in this country to the art of making the instruments upon which so many departments of medical science are more or less dependent. Mr. Spencer's labors and triumphs are well known. It is not so generally understood that excellent lenses have been made in this city. Mr. Alvan Clark, distinguished as an artist and as a maker of astronomical instruments, has employed his leisure, occasionally, in making objectives, several of which I have seen and found to compare very favorably with the best of the imported glasses of similar power. There has been little done as yet, however, in the way of providing the microscopist with those numerous accessories which he is constantly requiring, and which in London or Paris he can readily obtain. To get *very thin* glass, one must hunt up in New York, the American agency of Messrs. Chance, of Birmingham, which is to be found in an obscure warehouse remote from the common markets of scientific commodities. As for a set of delicate tests, it is doubtful if they can be had without importing them expressly. Some of Hett's and Topping's injected preparations may be had in New York, but only such as have been left after careful culling by others.

We shall have to find out that we can make many of these things for ourselves, which we are in the habit of importing; *all* of them, as soon as it will pay to make them. It would not be surprising to find, in ten years from this time, that there were more microscopists in America than in Europe. For here everybody must know something of everything; and as a microscope is *prima facie* evidence that the owner is a microscopist, it will become as necessary a part of the stock in trade as a stethoscope; which implies that the owner is a stethoscopist—even if he does not know which end to put to his ear, as once happened in a consultation in this region. Thus there will be growing up among us a market for microscopes and all that belongs to microscopic art, and the skill which has never failed to show itself whenever it has been called for, will find a new channel in providing for this want.

The art of *minute injection* has been, until of late, very little practiced in this country. Dr. Horner's preparations in the Wistar Museum, are among the most successful examples of it. The application of the achromatic microscope to the study of the tissues has given a fresh impulse to this branch of anatomical art, and many beautiful results have been obtained; such as we can hardly believe that Ruysch or Lieberkuhn can have approached, by what we know of their performances. Many of the injections of Berres and others, are figured in the work of Gerber; Hassall gives figures of those of several of the English anatomists; Dr. Neill, of Philadelphia, has given very beautiful representations of some of his own injections of the mucous membrane of the stomach. From these plates, those who have not access to the original preparations may form some idea of their delicacy and brilliancy.

Preparations of this kind, properly put up in preservative fluid, are of very great importance, especially to the teacher of microscopic art and science. It is in this capacity that I have had occasion to employ many such preparations, of some of which a few remarks will be here made.

The first I used were some made by or under the direction of Retzius, of Stockholm, lent me by Dr. Ware. One of these, an injection of the lobules of the liver, is a very beautiful exhibition of the two veins and the duct filled with different kinds of injections. They are put up in a somewhat rough way between two thick plates of glass.

The preparations of Mr. Hett, some of which were selected by Mr. Burnett, in London, and others purchased of the importer, are put up with great neatness, and on the whole the most brilliant specimens of minute injection of all those mentioned. They become infested with air-bubbles in the course of a year or two, which will in time require them to be taken out and the cells re-filled with fluid.

Those of Mr. Topping are injected in many cases with yellow instead of red, which makes them somewhat less showy than the others. They are, however, well filled and neatly mounted.

I have received from Dr. John Neill, of Philadelphia, specimens prepared by himself, the last received very perfect; the colored figures before referred to, which may be found in the American Journal of Medical Sciences, for Jan., 1851, show the delicacy of the injection and the use of such preparations in bringing out the nicer points of structure.

We have in this city a microscopist who has devoted himself with great assiduity and success to preparing and mounting specimens, many of which are injected by him with great nicety. Dr. Durkee, the gentleman referred to, has been his own instructor, and has succeeded, after many trials, in acquiring to a great extent the skill which is almost confined to a few persons abroad who make a business of preparing objects for microscopists. I will mention a few of these which I have seen, to give an idea of the points which they illustrate. Several of these which Dr. Durkee had the kindness to give me, I have used with much satisfaction in my demonstration.

1. Fœtal stomach, near cardiac orifice. A perfect injection, showing ridges, areolæ, but no villi.

2. Skin of the back of the hand, showing vascular net-work.

3. Mucous membrane of gall-bladder, finely injected, showing ridges, running into villi.

4. Membrana tympani injected, showing a non-vascular spot about the attachment of the handle of the malleus.

5. Malpighian corpuscles of the kidney in the human subject and in the ox, beautifully shown.

6. Tongue, showing the filiform papillæ, finely injected.

I have selected these as among the most successful preparations, but there are many others of much interest. Among the rest I should not forget the sections of bone, which Dr. Durkee has the art of making in a very superior way. I have made hundreds of them, and seen a great many made in this country and in Europe, but never saw more than one specimen equal to the best made by Dr. Durkee.

The injected preparations made in this country are apt to be inferior in color to the imported ones. The vermilion is not equal in brilliancy to that used by Mr. Hett. Once in a while it is found to contain specks which take off a little from the beauty of the specimen containing them. But it is evident that we are in the way of learning to do for ourselves what others have done for us, and there can be no doubt that the slight difficulties which stand in the way of absolute perfection, will be overcome as the principal ones have already been. It was said at the beginning of this communication, that the young practitioner had *time enough* to improve his knowledge of the microscope in his early years of practice. There are many hours which he must pass in his office, quite undisturbed, in company with his books and his thoughts. Let him add a microscope as a companion to these, and time will be wonderfully lightened for him, while he is acquiring the knowledge he will be very glad of in the busy years that are coming.

The microscope is of all philosophical instruments the most unfailing and untiring companion. The astronomer tells us that hardly more than a dozen nights in the year are adapted to his observations. He must watch all night exposed to cold and damp, surrounded by costly and cumbrous machinery. The microscopist sits down at his fireside or his window, with a little instrument before him, a mere toy to look at—a giant mightier than the slave of the lamp or the ring in its power of transformation. All that he wishes to observe upon, nature is ready to furnish him. Nothing is too precious or rare for him to covet; he wishes but a mere speck, a particle, such as the koh-i-noor could spare him. Nothing is repulsive, examined in its infinitesimal shape. The disease which infected the wards of a hospital does not betray itself in the narrow apartment where he studies all its intimate details. He may study and work until *practice* comes and takes him off his feet and floats him away into a world of other cares and duties, and year after year, every day will bring him something new to examine. I will say nothing of the utility, even the necessity of the microscope to the practical physician and the surgeon. As a mere illustrative companion to scientific study, as a mere intelligent plaything, it is the most precious gift to all who love to look at the universe as its inner life is revealed to the senses. To all who have done and are doing anything to render it more available for the purposes of study, we are under obligations which it is a pleasure to express, even if it is done as in this slight notice, which was suggested by the pleasure derived from examining the preparations made by Dr. Durkee.—*Boston Medical and Surgical Journal.*

